# Fcγ receptor binding is required for maximal immunostimulation by CD70-Fc

**DOI:** 10.3389/fimmu.2023.1252274

**Published:** 2023-10-27

**Authors:** Osman Dadas, Joel D. Allen, Sarah L. Buchan, Jinny Kim, H. T. Claude Chan, C. Ian Mockridge, Patrick J. Duriez, Anne Rogel, Max Crispin, Aymen Al-Shamkhani

**Affiliations:** ^1^ Antibody and Vaccine Group, Centre for Cancer Immunology, School of Cancer Sciences, Faculty of Medicine, University of Southampton, Southampton, United Kingdom; ^2^ Department of Molecular Biology and Genetics, Faculty of Arts and Sciences, European University of Lefke, Lefke, Cyprus; ^3^ School of Biological Sciences, Faculty of Environmental and Life Sciences, University of Southampton, Southampton, United Kingdom

**Keywords:** CD27, TNFRSF, costimulation, T cells, cancer, vaccine, immunotherapy

## Abstract

**Introduction:**

T cell expressed CD27 provides costimulation upon binding to inducible membrane expressed trimeric CD70 and is required for protective CD8 T cell responses. CD27 agonists could therefore be used to bolster cellular vaccines and anti-tumour immune responses. To date, clinical development of CD27 agonists has focussed on anti-CD27 antibodies with little attention given to alternative approaches.

**Methods:**

Here, we describe the generation and activity of soluble variants of CD70 that form either trimeric (t) or dimer-of-trimer proteins and conduct side-by-side comparisons with an agonist anti-CD27 antibody. To generate a dimer-of-trimer protein (dt), we fused three extracellular domains of CD70 to the Fc domain of mouse IgG1 in a ‘string of beads’ configuration (dtCD70-Fc).

**Results:**

Whereas tCD70 failed to costimulate CD8 T cells, both dtCD70-Fc and an agonist anti-CD27 antibody were capable of enhancing T cell proliferation *in vitro*. Initial studies demonstrated that dtCD70-Fc was less efficacious than anti-CD27 in boosting a CD8 T cell vaccine response *in vivo*, concomitant with rapid clearance of dtCD70-Fc from the circulation. The accelerated plasma clearance of dtCD70-Fc was not due to the lack of neonatal Fc receptor binding but was dependent on the large population of oligomannose type glycosylation. Enzymatic treatment to reduce the oligomannose-type glycans in dtCD70-Fc improved its half-life and significantly enhanced its T cell stimulatory activity *in vivo* surpassing that of anti-CD27 antibody. We also show that whereas the ability of the anti-CD27 to boost a vaccine response was abolished in Fc gamma receptor (FcγR)-deficient mice, dtCD70-Fc remained active. By comparing the activity of dtCD70-Fc with a variant (dtCD70-Fc(D265A)) that lacks binding to FcγRs, we unexpectedly found that FcγR binding to dtCD70-Fc was required for maximal boosting of a CD8 T cell response *in vivo*. Interestingly, both dtCD70-Fc and dtCD70-Fc(D265A) were effective in prolonging the survival of mice harbouring BCL1 B cell lymphoma, demonstrating that a substantial part of the stimulatory activity of dtCD70-Fc in this setting is retained in the absence of FcγR interaction.

**Discussion:**

These data reveal that TNFRSF ligands can be generated with a tunable activity profile and suggest that this class of immune agonists could have broad applications in immunotherapy.

## Introduction

Activation of conventional T cells requires T cell receptor (TCR) recognition of peptides bound to MHC molecules as well as signals delivered by costimulatory receptors interacting with their cognate ligands on antigen presenting cells. Costimulatory molecules enhance and sustain the magnitude of the signalling pathways downstream of the TCR/CD3 complex by recruitment of adaptor proteins and kinases. Ultimately, the combined signals emanating from the TCR/CD3 and costimulatory receptors lead to quantitative and qualitative changes that culminate in increased T cell proliferation, survival, metabolic fitness, and differentiation into effector cells (1, 2).

Costimulatory receptors primarily belong to either the immunoglobulin superfamily or the TNF receptor superfamily (TNFRSF), with both types of receptors contributing to the regulation of T cell immunity in a cell and infection specific manner (2). CD27 is a member of the TNFRSF expressed on almost all T cells, germinal centre and memory B cells, as well as a subset of NK cells (3). Earlier studies established an important role for CD27 in augmenting T cell responses in both humans and mice. Costimulation via CD27 was shown to play a complementary role to CD28 during the primary and secondary activation of CD8 T cells (4–7). In addition, CD27 costimulation was found to promote the development of CD4 Th1 T cells (8) and subsequently shown to exert suppressive effects on the function of Th17 cells (9). CD70, the CD27 ligand, is a homotrimeric type II transmembrane protein transiently upregulated on activated dendritic cells, B cells and T cells in response to CD40, Toll-like receptor or antigen receptor stimulation (6, 10–12).

The importance of the CD27-CD70 axis in human immunity was established with the discovery that individuals with inherited deficiency of either CD27 or CD70 have impaired CD8 T cell responses to Epstein-Barr virus (EBV) resulting in EBV-driven lymphoproliferation, hypogammaglobulinemia and lymphoma development (13–16). The non-redundant role of the CD27-CD70 axis in providing protection against EBV driven B cell malignancy suggests that enforced CD27 costimulation could potentially restore defective CD8 T cell-mediated immune surveillance of B cell tumours. To date, both agonist anti-CD27 antibodies and soluble forms of CD70 have been used to investigate the effect of enforced CD27 stimulation on T cell responses and anti-tumour activity (5, 17–21). However, there is no consensus on which of these agents represent the therapeutic of choice for delivering optimal CD27 costimulation. A wide range of agonistic activity has been reported for different anti-CD27 mAbs (21, 22). Furthermore, the activity of anti-CD27 mAbs may depend on antibody isotype which affects binding to the inhibitory and activatory Fcγ receptors (FcγRs) (18, 21). In contrast to anti-CD27 antibodies, soluble CD70 potentially offers an approach to deliver agonism without the need for FcγR mediated crosslinking. However, as soluble trimeric CD70 lacks biological activity, different approaches have been proposed to generate bioactive forms that form higher order oligomeric structures. One approach involved the attachment of the extracellular domain of CD70 to the C-terminus of human IgG1 Fc (5, 23). An alternative design to generate a more uniform hexameric protein comprising two adjacent trimeric CD70 proteins involved the attachment of three CD70 extracellular (ECD) domain fragments in a single chain format to the N-terminus of the Fc domain (20). Although these Fc fusion proteins were demonstrated to be functional, it was unclear if FcγR mediated hyper-crosslinking could further potentiate their stimulatory effects and therefore necessary for optimal activity. A soluble CD70 protein with a predictable activity profile could overcome the limitations of agonist anti-CD27 mAbs, but to date direct comparisons of the activity of soluble CD70 and anti-CD27 mAbs have not been reported.

Here we evaluate the *in vitro* and *in vivo* biological activity of soluble CD70 fusion proteins comparing them to agonist anti-CD27 mAb and identify key features that are required for optimal activity. Our data highlight the potential of CD70-based therapeutics as an alternative to agonist anti-CD27 mAbs.

## Materials and methods

### Generation of soluble CD70 fusion proteins and recombinant anti-CD27 antibody

Soluble trimeric CD70 (tCD70) was produced by fusing domains 3 and 4 of mouse CD4 to the CD4 to the ECD (S41-P195) of murine CD70. Briefly a DNA construct encoding a leader peptide (MEWSWVFLFFLSVTTGVHSEVQAHS), domains 3 and 4 of mouse CD4, a short linker (G3S) and the ECD of mouse CD70 was ordered commercially and supplied in the pcDNA3.1 expression plasmid. tCD70 was produced by transient transfection of 293F cells and purified from spent tissue culture supernatant by anti-CD4 affinity column chromatography 7 days after transfection (24). Soluble single-chain trimeric CD70 (sctCD70) was produced by fusing domains 3 and 4 of mouse CD4 via a G3S linker to three CD70 ECD (S41-P195) fragments separated by flexible linkers (G3S)_3_. The DNA construct was ordered commercially and supplied in pcDNA3.1. We also generated a dimer of trimer CD70-Fc fusion protein (dtCD70-Fc) by assembling three fragments encoding the ECD of mouse CD70 (S41-P195) separated by (G3S)_3_ linkers followed by the hinge and CH_2_/CH_3_ domains of mouse IgG1. The DNA fragment was excised from pcDNA3.1 with HindIII and EcoRI and subcloned into the expression vector pEE14 (Lonza), which was then transfected into suspension adapted Chinese hamster ovary cells (CHO-K1S) to generate stable lines. CHO-K1S cells expressing dtCD70-Fc were grown in a shaking incubator at 37°C and 8% CO_2_ in FortiCHO medium (Thermo Fisher) supplemented with methionine sulfoxamine, hypoxanthine and thymidine. The dtCD70-Fc protein was purified from 2-4 week spent tissue culture media by protein A column chromatography followed by preparative size exclusion chromatography (Superdex 200 26/950).

To produce anti-CD27 mouse IgG1, total RNA was extracted from the anti-CD27 hybridoma AT124-1 (17) and converted into cDNA using the SuperScript™ IV First-Strand Synthesis System (Thermo Fisher). Anti-mouse CD27 V_H_ and V_L_ sequences were amplified by PCR using degenerate 5’ primers and constant region specific 3’ primers. After verification by DNA sequencing, the V_H_ and V_L_ encoding DNA fragments were cloned in frame with the constant mouse heavy (IgG1) and light (kappa) chains, respectively, in pEE6.4 (Lonza). To generate stable CHO-K1S cell lines, the heavy and light chain expression cassettes in pEE6.4 were subcloned into a single expression plasmid (pEE12.4; Lonza) which was then transfected into CHO-K1S cells using GenePorter (Thermo Fisher).

### Affinity measurements by surface plasmon resonance

A Biacore T200 instrument and HBS-EP+ running buffer was used throughout (GE healthcare). Anti-human IgG was first attached to the CM5 chip by amine coupling following the manufacturer protocol (GE healthcare). Recombinant mouse CD27-human Fc (R&D systems) was then captured for 1 min at a flow rate of 10 μl/min. The flow rate was then increased to 30 μl/min before injection of serially diluted CD70 fusion proteins. The chip was regenerated with injection of MgCl_2_ (3 M) for 1 min at flow rate 20 μl/min. The *k*_a_ and *k*_d_ were determined using the Biacore Bioevaluation software and the K_D_ values were calculated as *k*_a_/*k*_d_.

To examine the binding of dtCD70-Fc and anti-CD27 mAb to FcRn, ~2000 response units of dtCD70-Fc or anti-CD27 were immobilized onto a CM5 chip via amine coupling. Serially diluted recombinant mouse FcRn (R&D systems) was injected for 3 min at a flow rate of 30 μl/min in HBS-EP+ buffer adjusted to pH 6. The chip was regenerated with injection of HBS-EP+ (pH 7.4). The K_D_ values were calculated using steady-state binding levels at different concentrations of FcRn.

### Glycosylation analysis

Glycoproteins (50 µg) were subjected to proteolytic digestion with trypsin. Before digestion, samples were denatured, reduced and alkylated by incubation for 1 h at room temperature (RT) in a 50 mM Tris/HCl, pH 8.0 buffer containing 6 M urea and 5 mM dithiothreitol, followed by addition of 20 mM iodacetamide for a further 1 hr at RT in the dark, and then additional dithiothreitol (20 mM) for another 1 hr, to eliminate any residual iodoacetamide. The alkylated samples were buffer exchanged into 50 mM Tris/HCl, pH 8.0 using Vivaspin columns (GE healthcare). Trypsin (1.7 µg) was added to glycoproteins (50 µg) and the mixture incubated at 37°C for 16 h. Trypsin was heat inactivated and glycopeptides were extracted using C18 Zip-tip (Merck Millipore) following the manufacturers protocol.

The peptides were dried, re-suspended in 0.1% formic acid and analyzed by nanoLC-ESI MS with an Easy-nLC 1200 (Thermo Fisher Scientific) system coupled to a Fusion mass spectrometer (Thermo Fisher Scientific) using higher energy collision-induced dissociation (HCD) fragmentation. Peptides were separated using an EasySpray PepMap RSLC C18 column (75 µm × 75 cm). A trapping column (PepMap 100 C18 3μM 75μM x 2cm) was used in line with the LC prior to separation with the analytical column. The LC conditions were as follows: 275 min linear gradient consisting of 0-32% acetonitrile in 0.1% formic acid over 240 minutes followed by 35 minutes of 80% acetonitrile in 0.1% formic acid. The flow rate was set to 300 nl/min. The spray voltage was set to 2.7 kV and the temperature of the heated capillary was set to 40°C. The ion transfer tube temperature was set to 275°C. The scan range was 400−1600 m/z. The HCD collision energy was set to 50%, appropriate for fragmentation of glycopeptide ions. Precursor and fragment detection were performed using an Orbitrap at a resolution MS1 = 100,000. MS2 = 30,000. The AGC target for MS1 = 4e5 and MS2 = 5e4 and injection time: MS1 = 50ms MS2 = 54ms.

Data analysis and glycopeptide identification were performed using Byonic (Version 2.7) and Byologic software (Version 2.3; Protein Metrics Inc.). The glycopeptide fragmentation data were evaluated manually for each glycopeptide; the peptide was scored as true-positive when the correct b and y fragment ions were observed along with oxonium ions corresponding to the glycan identified. The MS data were searched using the Protein Metrics 305 N-glycan library with sulfated glycans added manually. The relative amounts of each glycan at each site as well as the unoccupied proportion were determined by comparing the extracted chromatographic areas for different glycotypes with an identical peptide sequence. All charge states for a single glycopeptide were summed. The precursor mass tolerance was set at 4 ppm and 10 ppm for fragments. A 1% false discovery rate (FDR) was applied. The relative amounts of each glycan at each site as well as the unoccupied proportion were determined by comparing the extracted ion chromatographic areas for different glycopeptides with an identical peptide sequence. Glycans were categorized according to the composition detected. Any composition containing HexNAc(2)Hex(>3) was classified as oligomannose-type, those containing at least one fucose and/or sialic acid were classified as Fucose or NeuAc respectively. Any composition containing Hex(3) was classified as ‘Hex(3), no galactose’. GlcNAc(1)/GlcNAc(1)Fuc(1) is included as a separate category to highlight the remnant monosaccharide resulting from endoglycosidase H (Endo H) treatment.

### Endo H treatment

Typically, 20 mg of dtCD70-Fc were incubated with 100000 units of Endo H in acetate buffer (0.1M, pH 5.2) at 37°C for 4 h. The optimal enzyme/substrate ratio determined by digestion trials. dtCD70-Fc was then dialysed against phosphate buffered saline and re-purified by size-exclusion chromatography.

### T cell proliferation assay

Single cell suspensions were prepared from the spleens of C57BL/6 mice. Following lysis of red blood cells, splenocytes (2 x 10^5^) in U-bottom shaped 96-well plates were stimulated with soluble anti-CD3 mAb (clone 145-2C11, prepared in-house) and additionally with CD70 proteins, anti-CD27 mAb or control mouse IgG1 (anti-human CD16 clone 3G8, prepared in-house) at the concentrations indicated in the Figure legends. Cells were incubated in a final volume of 200 μl at 37°C and 5% CO_2_ in a humidified incubator for 48 h and then 1 μCi/well of ^3^H-thymidine was added for an additional 17 h before harvesting. The cells were then lysed using a harvesting system and lysates transferred to filter plates (Opti-plate-96, Perkin Elmer). Scintillant fluid (40 μl/well) (Perkin Elmer) was added and incorporation of ^3^H-thymidine into proliferating cells was measured using a β-emission counter.

### NFκB reporter assay

The Jurkat NFκB GFP reporter cell line (System Biosciences) was transfected using Lipofectamine 2000 (Thermo Fisher Scientific) with pcDNA3.1 encoding mouse CD27 cDNA and stable clones were then selected using 1 mg/ml geneticin. To study NFκB activation, cells were incubated with fusion proteins or anti-CD27 mAb for 6 h at 37°C and the magnitude of NFκB activation was measured by detection of GFP production using flow cytometry. In some experiments Jurkat cells were co-cultured with CHO-K1 cells stably expressing mouse FcγRIIB (provided by Dr Hannah Smith and Prof Mark Cragg, University of Southampton).

### Endotoxin detection

Recombinant proteins were regularly assessed for endotoxin levels using the Endosafe-PTS portable test system (Charles River, Massachusetts, USA) and found to contain < 5 EU per mg protein.

### *In vivo* experiments

Mice (C57BL/6, BALB/c, OT-I and FcγR1,2,3,4 null) were maintained in the Biomedical Research Facility unit of University of Southampton. Mice were kept on a 12 h light/dark cycle, provided with environmental enrichment and the temperature was maintained between 20-24°C. OT-I TCR transgenic mice specific for the ovalbumin (OVA)-derived peptide 257-264 (OVA_257-264_) (25) and FcγR1,2,3,4 deficient mice (generated by Dr Sjef Verbeek (26)) have been established previously. All experiments were conducted under UK Home Office licence numbers PA4C79999 and IE7C34E6C and following approval by the local ethics committee, reporting to the Home Office Animal Welfare Ethical Review Board (AWERB) at the University of Southampton. Age (8-12 weeks) and sex matched experimental animals were maintained in individually ventilated cages and food and water was available *ad libitum*. Mice were visually checked daily if adverse effects were anticipated or if mice were nearing a humane end point.

To determine the effect of CD27 agonists on T-cell activation *in vivo*, total leukocytes prepared from the spleens of OT-I mice were adoptively transferred into C57BL/6 recipients. In some experiments congenic OT-I mice expressing the CD45.1 allele were utilised. Mice were rested for 24 h before challenge with OVA_257-264_ peptide (30 nmol) in combination with 250 μg control mouse IgG1, dtCD70-Fc variants or anti-CD27 mAb as described in the Figure legends. The number of transferred T cells was determined by PE-labelled H-2K^b^ OVA_257-264_ tetramers and then adjusted to achieve the desired numbers. When assessing the role of FcγRs *in vivo*, CD8^+^ T cells from OT-I mice were first purified using CD8α MicroBeads to remove FcγR expressing accessory cells (Miltenyi Biotec) prior to adoptive transfer into FcγR1,2,3,4 null mice. OT-I T cell expansion in recipient mice was monitored by peripheral blood sampling and flow cytometry. To assess the endogenous OVA_257-264_ CD8^+^ T cell response, mice were injected i.v. with OVA protein (Sigma-Aldrich) in combination with antibodies or dtCD70-Fc and subsequently received 2 further i.v. injections of antibodies or dtCD70-Fc as described in the Figure legends.

For tumour immunotherapy experiments, groups of age-matched mice were injected i.v. with 5x10^6^ BCL_1_ B cell lymphoma (17, 27, 28) on day 0 followed by CD27 agonist proteins on days 5, 6, 7 and 8 post tumour inoculation (200 µg/d). Survival period to the humane end point was plotted using the Kaplan-Meier method with analysis for significance by the log-rank (Mantle-Cox) test.

Serum concentrations of dtCD70-Fc proteins and anti-CD27 mAb after i.v. injection were measured by ELISA. For dtCD70-Fc, we used an anti-CD70 mAb (6) for capture and horseradish peroxidase-conjugated rat anti-mouse IgG (Jackson Immunoresearch) for detection. To determine the concentration of anti-CD27, we used CD27-Fc (R&D Systems) as a capture reagent and horseradish peroxidase-conjugated goat anti-mouse IgG for detection.

### Flow cytometry

Antibodies used for staining were purchased from eBioscience: anti-CD8α-APC (53-6.7), anti-CD62L-eFluor450 (MEL-14), anti-CD45.1-eFluor450 (A20) and anti-CD44-FITC (IM7). The numbers of adoptively transferred OT-I T cells were checked by staining with PE-labelled H-2K^b^ OVA_257-264_ tetramers and their naïve phenotype confirmed by CD62L/CD44 staining (~95% CD62L high and CD44 low). Throughout, a blocking anti-FcγRII/III antibody (2.4G2; 10 μg/ml) was added to cells for 15 minutes at 4°C prior to incubation with surface staining antibodies for 30 minutes at 4°C. Red blood cells were then lysed and cells were washed prior to analysis on a BD FACS Canto II using the BD FACSDiva software.

### Statistical analysis

Statistical analyses were performed using GraphPad Prism software (9.4.1). Statistical analyses of pairwise comparisons are by two-tailed, non-paired Students t test and for multiple comparisons by one-way or two-way ANOVA with Tukey’s *post hoc* multiple comparisons test, as appropriate. p < 0.05 is considered significant throughout. N numbers are defined in the relevant legends. Statistical comparisons between survival to the humane end point are by Log-rank test, and again statistical significance is considered at p < 0.05. P values are indicated in the figure legends; *p<0.05, **p<0.01, ***p<0.001, ****p<0.0001.

## Results

### Generation and *in vitro* activity of soluble CD70 proteins

**Figure 1:**
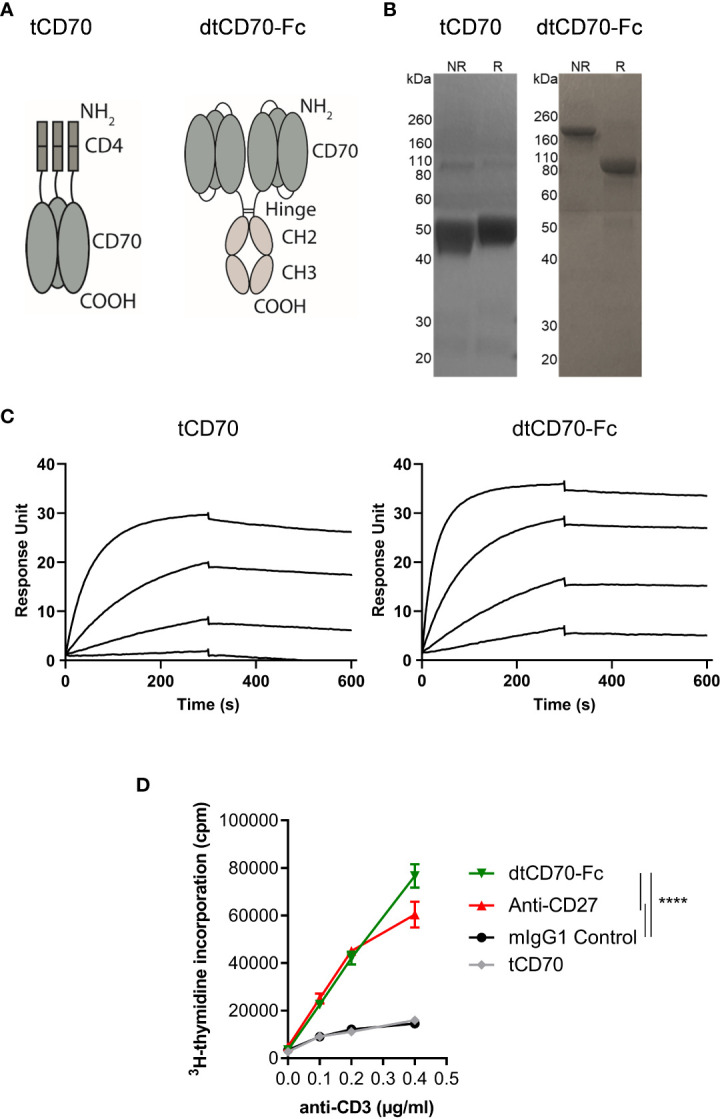
Structure, receptor binding profile, and *in vitro* T cell costimulatory effects of CD70 fusion proteins. **(A)** Schematic representation of tCD70 and dtCD70-Fc fusion proteins. **(B)** Purified tCD70 and dtCD70-Fc proteins (5 µg) were analysed using a 10% SDS-polyacrylamide gel under non-reducing (NR) or reducing (R) conditions. The gel was stained with Coomassie blue. **(C)** Overlay of SPR sensograms demonstrating binding of CD70 fusion proteins (1.56, 6.25, 25 and 100 nM) to captured recombinant mouse CD27 and their subsequent dissociation. **(D)** Splenocytes were stimulated for 72 h with various concentrations of soluble anti-CD3 and the indicated proteins (10 μg/ml). Proliferation of T cells as assessed by measurement of [^3^H]-thymidine incorporation. Data points are the mean of triplicate measurements +/- SE and the data are representative of two independent experiments. Statistical comparisons at the highest anti-CD3 concentration are indicated. **** P < 0.0001, two-way ANOVA with Tukey’s multiple comparison test.

tCD70 was produced by fusing the murine CD70 ECD, which naturally trimerises, to a monomeric tag that consists of domains 3 and 4 of mouse CD4 (**Figure 1A**). A similar approach was used previously to produce soluble trimeric OX40 and CD30 ligands (24, 29). dtCD70-Fc was produced by fusing three copies of the murine CD70 ECD in a single chain format to the hinge-CH_2_-CH_3_ domains of mouse IgG1 (**Figure 1A**). Proteins were purified by mAb affinity (tCD70) or protein A (dtCD70-Fc) column chromatography and subsequently by size-exclusion chromatography. The observed molecular weights (MW) of the single polypeptide chains under reducing conditions were consistent with the predicted MW of 51.5 kDa and104.3 kDa for the CD4 and Fc fusion proteins, respectively (**Figure 1B**). A higher MW band for dtCD70-Fc was observed under non-reducing conditions verifying the presence of a disulfide-linked Fc dimer (**Figure 1B**). Furthermore, protein purity and the absence of protein aggregates were confirmed by analytical size-exclusion chromatography (**Supplementary Figure 1**). To further confirm the integrity of the CD70 proteins we used SPR analysis to assess the binding to CD27. The CD70 proteins showed similar association and dissociation profiles and bound to immobilized mouse CD27 protein with an apparent affinity of 1.2 nM and 0.6 nM, respectively (**Figure 1C**). To assess the immune stimulatory activity of the soluble CD70 proteins, we examined their effects on T cell proliferation by measurement of cellular [^3^H]-thymidine incorporation. Stimulation of splenic cells with sub-optimal concentrations of anti-CD3 resulted in limited cell proliferation, which was not enhanced by the addition of tCD70, consistent with previous findings (23). In contrast, the addition of either agonist anti-CD27 mAb (17, 30) or dtCD70-Fc resulted in 3-4-fold increase in T cell proliferation (**Figure 1D**). To examine if crosslinking could potentiate the activity of tCD70, we developed an assay that utilises human Jurkat cells engineered to express mouse CD27 and a GFP reporter of NFκB activation. Crosslinking of tCD70 was then attempted using an anti-mouse CD4 mAb that binds to the CD4 tag on the tCD70 protein, but this did not result in increased NFκB activity (**Supplementary Figure 2A**). We reasoned that the presence of 3 copies of the CD4 tag in tCD70 interfered with the ability of the anti-CD4 mAb to crosslink tCD70 and therefore generated a single-chain tCD70 protein (sctCD70) with a single CD4 tag (**Supplementary Figures 2B-D**). Although crosslinking did enhance the activity of sctCD70, the magnitude of NFκB activation as determined by GFP expression was substantially lower than that achieved using dtCD70-Fc (**Supplementary Figure 2E**). Given that soluble CD70 in its trimeric form lacks bioactivity and that our study is mainly concerned with developing agents suitable for *in vivo* applications, we decided to focus on the dtCD70-Fc protein and compare its activity with anti-CD27 mAb.

### dtCD70-Fc induces expansion of T cells *in vivo*

**Figure 2:**
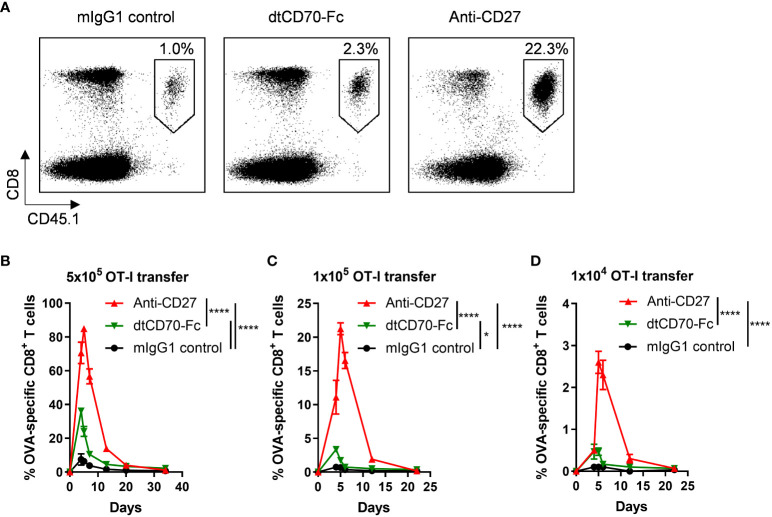
Effects of anti-CD27 mAb and dtCD70-Fc on OT-I T cell expansion *in vivo*. OT-I TCR transgenic T cells were adoptively transferred into C57BL/6 recipients. Mice were then immunised by i.v. injection of OVA_257-264_ in combination with control mouse IgG1 (mIgG1), dtCD70-Fc or anti-CD27. The next day mice received an additional dose of mIgG1, dtCD70-Fc or anti-CD27. Antigen specific CD8^+^ T cells in peripheral blood were enumerated at the indicated time points by staining with anti-CD8α and anti-CD45.1 **(A, C, D)** or anti-CD8α and OVA_257-264_ tetramer **(B)**. **(A)** Representative dot plots showing the percentage of OVA specific CD8^+^ T cells out of lymphocytes at the peak of the response (day 5). **(B–D)** Expansion of OVA specific CD8^+^ T cells after adoptive transfer of different numbers of OT-I T cells plotted as percentage out of lymphocytes. Data points represent the mean +/- SE (n = 3 mice/group). * P < 0.05, **** P < 0.0001, two-way ANOVA with Tukey’s multiple comparison test.

We assessed the activity of the dtCD70-Fc protein in the OT-I adoptive transfer model. We adoptively transferred different numbers of OT-I CD8 T cells into recipient mice and then challenged them with OVA_257-264_ peptide. Three groups of mice were then given either irrelevant mouse IgG1 as a control, agonist anti-CD27 mAb or dtCD70-Fc. Although agonist anti-CD27 mAb and dtCD70-Fc were both able to boost OT-I T cell expansion when compared with the IgG1 control, the magnitude of OT-I T cell expansion in the dtCD70-Fc group was significantly lower than that in the agonist anti-CD27 mAb group (**Figure 2**). Furthermore, when the frequency of adoptively transferred OT-I T cells was reduced, the difference in activity between the agonist mAb and dtCD70-Fc became more pronounced (**Figure 2**). Thus, at OT-I frequencies approaching physiological levels, dtCD70-Fc induced ~6-7 fold less T cell expansion compared with the anti-CD27 mAb. Since dtCD70-Fc and agonist anti-CD27 mAb were similarly able to stimulate T cell proliferation in cultures of splenocytes, we reasoned that the differences in the observed activity *in vivo* might be due to faster plasma clearance of the dtCD70-Fc protein.

### Oligomannose-type glycans contribute to reduced persistence of dtCD70-Fc in the circulation

**Figure 3:**
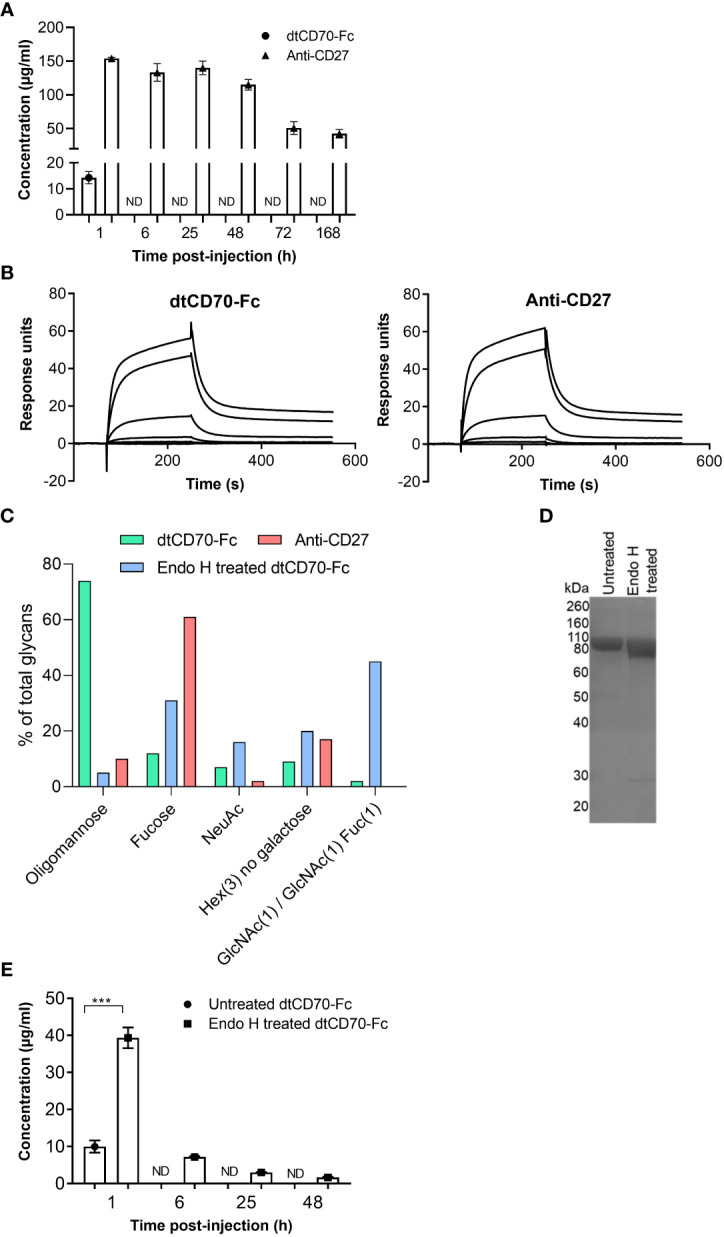
Oligomannose-type glycans in dtCD70-Fc contribute to its short half-life *in vivo*. **(A)** The concentrations of dtCD70-Fc and anti-CD27 mAb were determined in serum samples by ELISA at the indicated intervals following i.v. injection of proteins (250 μg). **(B)** Overlay of SPR sensograms demonstrating binding of FcRn at different concentrations (0, 0.8, 4, 20, 100, 500 nM) to dtCD70-Fc or antiCD27 immobilized directly onto a CM5 sensor chip. **(C)** Site-specific glycan analysis of dtCD70-Fc with and without Endo H treatment and anti-CD27 mAb. Bar graphs represent the average relative abundance of glycans detected across all sites on the molecule. Any composition containing HexNAc(2)Hex(>3) was classified as oligomannose-type, those containing at least one fucose and/or sialic acid were classified as Fucose or NeuAc respectively. Any composition containing Hex(3) was classified as “Hex(3), no galactose”. GlcNAc(1)/GlcNAc(1)Fuc(1) is included as a separate category to highlight the remnant saccharides resulting from Endo H treatment. **(D)** Analysis of oligomannose digestion by SDS-PAGE. Untreated dtCD70-Fc or an aliquot of the Endo H reaction (~ 5 μg protein) was run on a 10% SDS-polyacrylamide gel under reducing conditions. Proteins were revealed by Coomassie blue staining. **(E)** The concentrations of untreated or Endo H treated dtCD70-Fc were determined in serum samples by ELISA at the indicated intervals after i.v. injection of proteins (250 μg). Data points represent the mean +/- SE (n = 3 mice/group) and are representative of two independent experiments. *** P < 0.001, unpaired two-tailed t test.

We measured the serum concentrations of anti-CD27 mAb and dtCD70-Fc in the circulation over a period of 7 days and found that in contrast with anti-CD27 mAb, dtCD70-Fc was rapidly cleared from the circulation (**Figure 3A**). The serum concentration of dtCD70-Fc 1 h after i.v. administration was approximately one tenth that of the anti-CD27 mAb and was below 0.5 μg/ml by 6 h (**Figure 3A**). The neonatal Fc receptor (FcRn) within the acidic endosomal compartment binds to the Fc domain and facilitates recycling to the cell surface leading to the observed long circulatory half-lives of antibodies (31). Given the poor persistence of the dtCD70-Fc protein, we speculated that attachment of the CD70 ECD to the Fc domain could have reduced binding to FcRn. However, assessment of the dtCD70-Fc interaction with mouse FcRn by SPR showed that binding remained intact (**Figure 3B**), and the affinity of the interaction was similar to that of anti-CD27 mouse IgG1 binding to mouse FcRn (K_D(dtCD70-Fc)_ = 4.6 x 10^-8^ M; K_D(anti-CD27)_ = 4.9 x 10^-8^ M).

The dtCD70-Fc protein is predicted to be heavily glycosylated due to the presence of 10 potential N-linked glycan sites in each of its polypeptide chains. Nine of the N-linked glycosylation sites are found in the CD70 part (3 in each of the CD70 ECDs) with the remaining site present in the CH_2_ domain of the Fc. In contrast, the anti-CD27 mAb contains the canonical N297 glycosylation site in the Fc region as well as an additional site in the variable domain of the heavy chain (N59). Given that the type of N-glycan can have a major impact on the plasma half-life of glycoproteins (32–34), we performed site-specific glycan analysis of dtCD70-Fc and anti-CD27 mAb by liquid chromatography-mass spectrometry. This analysis revealed that on average 74% of the total glycans present in dtCD70-Fc were oligomannose-type with Man5-9 representing the major (95%) forms, whereas the figure for anti-CD27 mAb was 10% (**Figure 3C**). As the presence of oligomannose-type glycans is known to accelerate the clearance of glycoproteins, including antibodies, via uptake by the mannose receptor (32–35), we investigated if enzymatic removal of oligomannose-type glycans with Endo H could improve the persistence of dtCD70-Fc. Reduction of oligomannose-type glycans following Endo H treatment of dtCD70-Fc was confirmed by glycan analysis using liquid chromatography-mass spectrometry (**Figure 3C**, **Supplementary Figure 3**) as well as by SDS-PAGE which demonstrated increased mobility of the partially deglycosylated dtCD70-Fc (**Figure 3D**). Concurrent with the reduction in oligomannose-type glycans, there was an increase in N-acetylglucosamine (GlcNAc) and/or GlcNAc-fucose (**Figure 3C**), consistent with Endo H mediated cleavage between the two GlcNAc residues in the core region. Overall, although Endo H treatment of dtCD70-Fc reduced the number of N-linked glycans that contain oligomannose, there were still more oligomannose-containing glycans per dtCD70-Fc molecule after Endo H treatment compared with the anti-CD27 mAb (**Supplementary Figure 3**). To assess the effect of the reduction in the abundance of oligomannose-type glycans on the persistence of dtCD70-Fc *in vivo*, we compared the plasma half-lives of the two dtCD70-Fc proteins and found that removal of oligomannose-type glycans resulted in delayed clearance (**Figure 3E**). These results identify oligomannose-type glycans as important mediators of the rapid *in vivo* clearance of dtCD70-Fc and highlight a potential approach to improve bioactivity.

### Glycan trimming converts dtCD70-Fc into a potent agonist *in vivo*

**Figure 4:**
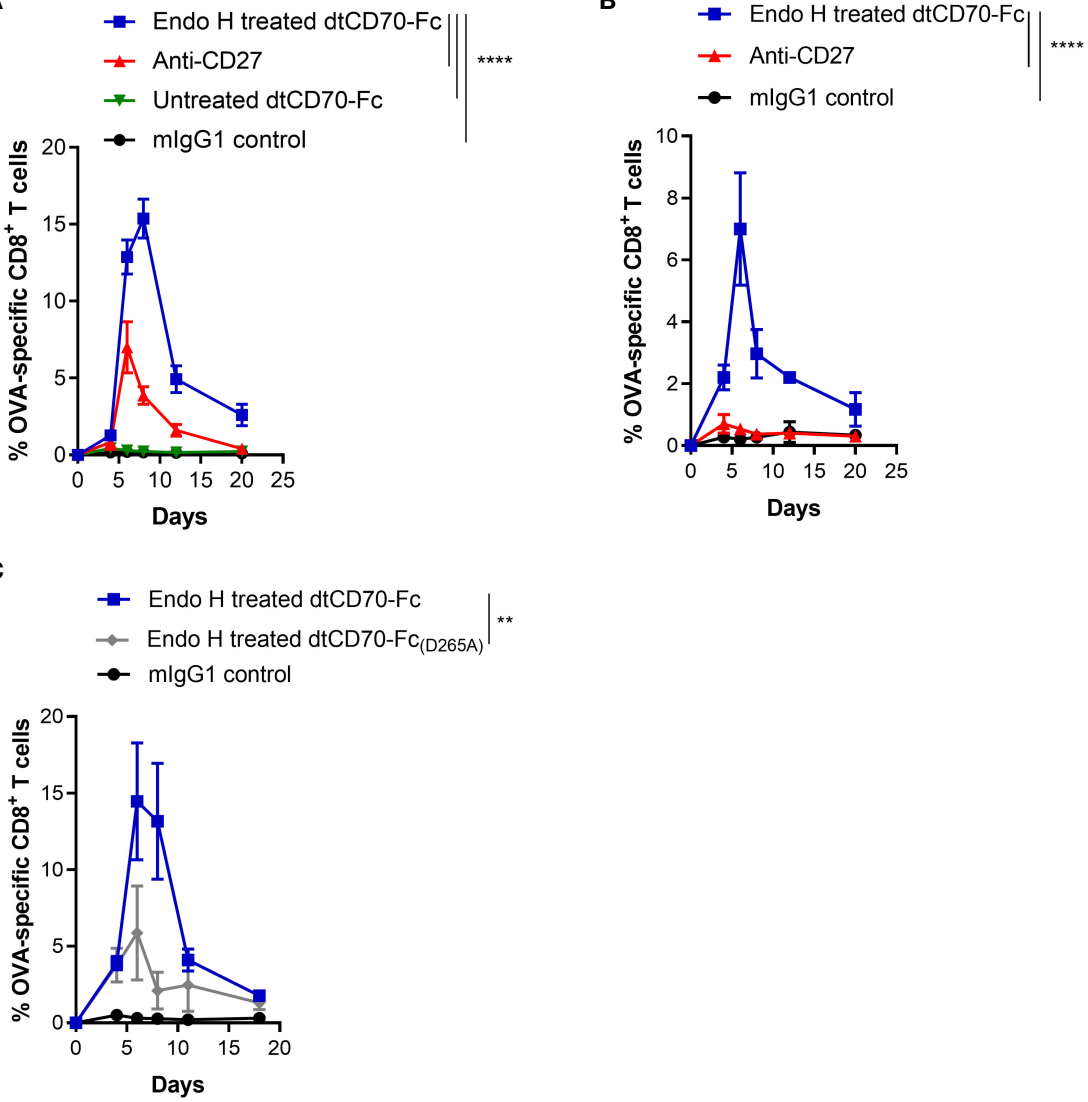
Glycan trimming and FcγR binding potentiate the immunostimulatory activity of dtCD70-Fc *in vivo*. **(A)** Purified OT-I CD45.1^+^ congenic CD8^+^ T cells (1 x 10^4^) were adoptively transferred into C57BL/6 recipients. Mice were then immunised with OVA_257-264_ in combination with control mIgG1, dtCD70-Fc, Endo H treated dtCD70-Fc or anti-CD27. The next day mice received an additional dose of mIgG1, dtCD70-Fc, Endo H treated dtCD70-Fc or anti-CD27. Antigen specific CD8^+^ T cells in peripheral blood were enumerated at the indicated time points and data are presented as percentage OVA-specific CD8^+^ T cells out of total CD8^+^ T cells. **(B)** *In vivo* agonistic activity of Endo H treated dtCD70-Fc in FcγR null mice. Adoptive transfer of OT-I T cells and immunisation was carried out as in **(A)** except that the recipient mice were FcγR null. **(C)** Comparison of the agonistic activity of Endo H treated dtCD70-Fc and dtCD70-Fc_(D265A)_ proteins. Purified OT-I T cells were adoptively transferred into C57BL/6 recipients and mice were immunised as indicated in **(A)**. Data points represent the mean +/- SE (n = 3 mice/group) and are representative of two independent experiments. ** P < 0.01, **** P < 0.0001, two-way ANOVA with Tukey’s multiple comparison test.

An initial assessment of the costimulatory effects of dtCD70-Fc demonstrated that Endo H treatment did not significantly alter its ability to enhance T cell proliferation *in vitro* (**Supplementary Figure 4**). Next, we investigated if the improved half-life of Endo H treated dtCD70-Fc would translate into improved bioactivity *in vivo*. We used a vaccination model wherein adoptive transfer of low numbers of OT-I T cells and injection of unmanipulated dtCD70-Fc gave a minimal T cell response. **Figure 4A** shows that OT-I expansion was markedly enhanced following injection of Endo H treated dtCD70-Fc, leading to levels of T cell expansion that surpassed that observed with agonistic anti-CD27 mAb. Similarly, the endogenous OVA_257-264_ specific CD8 T cell response was significantly enhanced after administration of dtCD70-Fc compared to anti-CD27 mAb (**Supplementary Figure 5**).

Several studies have shown that the agonistic activity of antibodies targeting various members of the TNFRSF are dependent on antibody hyper-crosslinking mediated by antibody binding to FcγRs, especially inhibitory FcγRIIB (36–38). Consistent with previous findings, the agonistic activity of the anti-CD27 mAb (mouse IgG1) was significantly diminished when OT-I T cells were adoptively transferred into FcγR deficient recipient mice (**Figure 4B**). In contrast, Endo H treated dtCD70-Fc was still able to induce OT-I T cell expansion in the absence of FcγRs, suggesting that FcγR-mediated dtCD70-Fc hyper-crosslinking is not essential for its activity (**Figure 4B**). Although dtCD70-Fc was clearly active in FcγR deficient mice, the magnitude of the OT-I T cell response was lower than that reached in the FcγR sufficient mice (**Figures 4A, B**). To further explore the possibility that the activity of dtCD70-Fc may have been potentiated by binding to FcγRs, we first confirmed that Endo H treated dtCD70-Fc is capable of binding to FcγRIIB and FcγRIII (**Supplementary Figure 6**), consistent with the binding specificity of mouse IgG1 Fc to murine FcγRs (36). Next, we introduced a mutation (D265A) in the CH_2_ domain known to abolish binding to mouse FcγRs without affecting half-life (39) and then compared the activity of Endo H treated dtCD70-Fc_(D265A)_ with the FcγR competent dtCD70-Fc. **Figure 4C** shows that while both dtCD70-Fc and dtCD70-Fc_(D265A)_ were able to stimulate OT-I T cell expansion, the presence of the wild-type Fc domain resulted in a 3-fold higher OT-I T cell expansion during the primary response. Further, we confirmed that introduction of the D265A mutation did not have a detrimental effect on the half-life as both Endo H treated dtCD70-Fc and dtCD70-Fc_(D265A)_ were similarly cleared from the circulation (**Supplementary Figure 7**, **Figure 3E**). Lastly, we confirmed that although functional as a soluble protein, the activity of dtCD70-Fc was enhanced when Jurkat NFκB-GFP reporter cells expressing mouse CD27 were co-cultured with FcγRIIB expressing cells (**Supplementary Figure 8**). Thus, taken together these findings support the notion that although not essential for activity, the interaction with FcγRs may be desirable for maximising the potency of dtCD70-Fc.

**Figure 5:**
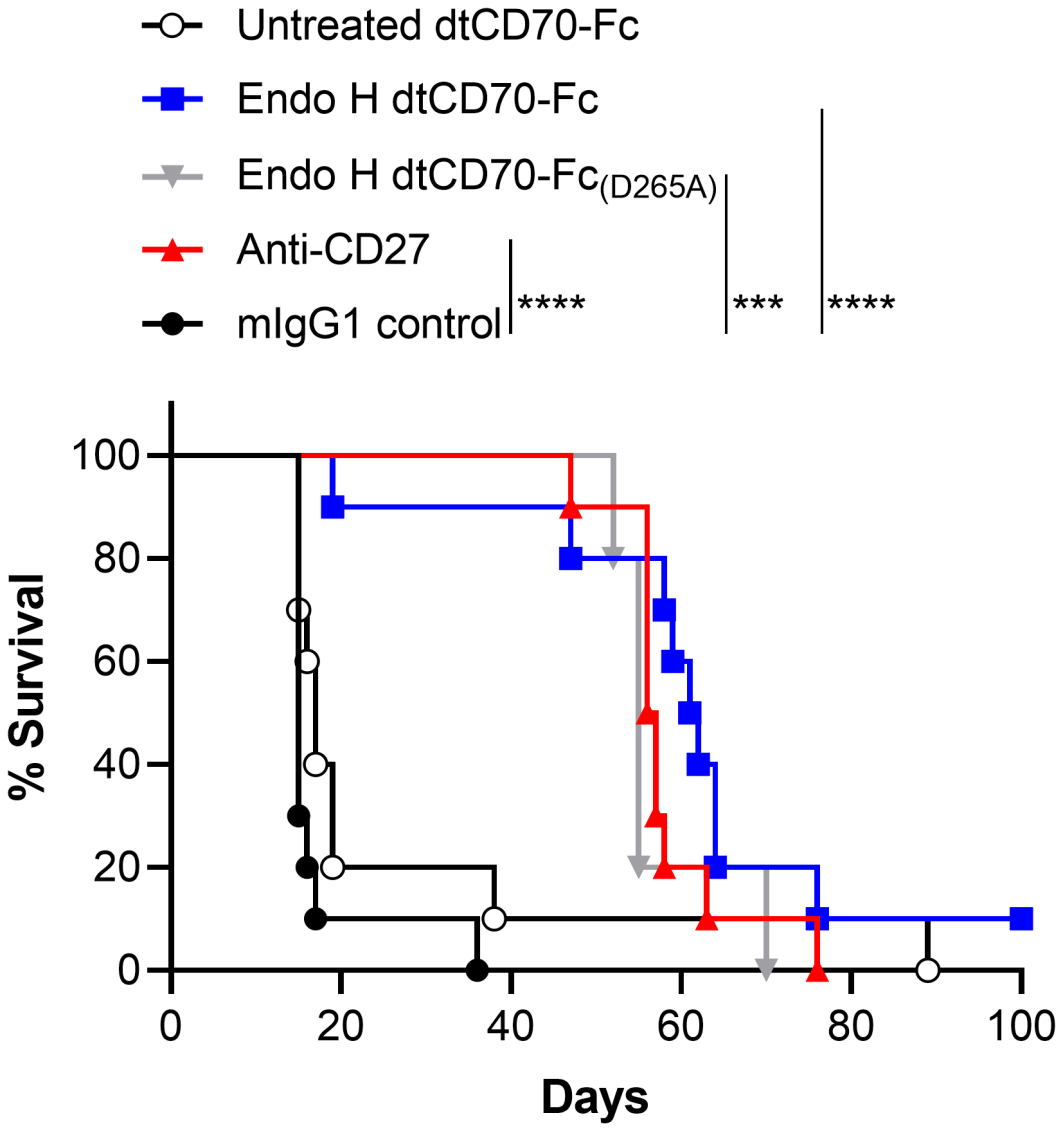
Therapeutic activity of dtCD70-Fc against BCL_1_ lymphoma. Groups of mice received 5 x 10^6^ BCL_1_ cells i.v. on day 0 and then mIgG1 control, dtCD70-Fc, Endo H treated dtCD70-Fc, Endo H treated CD70-Fc_(D265A)_ or anti-CD27 on days 5, 6, 7 and 8 (200 μg/d). Mice were monitored for tumour development and survival to the humane end point was plotted using the Kaplan-Meier method. *** P < 0.001, **** P < 0.0001, log-rank (Mantle-Cox) test (n = 5 - 10 mice/group).

Finally, we evaluated the therapeutic activity of dtCD70-Fc against the BCL_1_ lymphoma, a transplantable B cell tumour that originally arose spontaneously in a BALB/c mouse (27, 28). BCL_1_ lymphoma, which primarily develops in the spleen of recipient mice, is supressed by anti-CD27 mouse IgG1, an isotype that lacks effector function (ADCC and ADCP), consistent with the CD8 T cell stimulatory effects delivered by this isotype (18). Administration of anti-CD27 mAb, Endo H treated dtCD70-Fc or Endo H treated dtCD70-Fc_(D265A)_ significantly prolonged the survival of BCL1-bearing mice when compared to the mouse IgG1 control group (**Figure 5**). In contrast, administration of dtCD70-Fc with a large population of oligomannose-type glycosylation (untreated with Endo H) did not lead to statistically significant improvement in survival, consistent with lesser ability of this protein to stimulate expansion of OT-I T cells *in vivo* (**Figure 4A**). The median survival of mice given anti-CD27 mAb, Endo H treated dtCD70-Fc and Endo H treated dtCD70-Fc_(D265A)_ was 56.5, 61.5 and 55 days, respectively, which compared favourably with a median survival of 15 days in the control group. Overall, the data demonstrate that a substantial part of the dtCD70-Fc activity is retained in the absence of FcγR binding.

## Discussion

The overarching aim of the current study was to develop a potent CD27 agonist suitable for *in vivo* application. Current efforts to develop agonist antibodies targeting CD27 as well as other members of TNFRSF have been fraught with difficulties due to the vastly different immunostimulatory activities displayed by agents targeting the same receptor (21, 22, 40). Agonism is known to correlate with the ability of antibodies to induce receptor clustering, an attribute that is affected by epitope, antibody hinge flexibility, interaction with FcγRs, and affinity (18, 21, 36, 40–42). Furthermore, although co-engagement of FcγRIIB by anti-TNFRSF antibodies has been shown to promote agonism *in vivo* (36–38), this approach is highly sensitive to levels of FcγRIIB expression which vary depending on the tissue and cellular source (43).

Here we have evaluated an alternative approach that could overcome some of the limitations associated with antibody-based agonists. Given that soluble tCD70 fails to costimulate T cells despite high affinity binding to CD27 (**Figure 1** and (23)), we opted to generate a protein with two adjacent trimeric CD70 units, wherein 3 extracellular CD70 fragments were fused to the hinge-CH_2_CH_3_ domains of mouse IgG1 in a single chain format (dtCD70-Fc). dtCD70-Fc provided potent T cell costimulation signals culminating in increased T cell proliferation, demonstrating that forced dimerization of CD70 trimers is required for activating CD27 signalling (**Figure 1**). Members of the TNFRSF can be subdivided into those that are effectively activated by trimeric ligands (category I) and others that require further oligomerisation (category II) to facilitate downstream assembly and activation of the signalosome (44). Our findings showing that dtCD70-Fc, but not tCD70, was able to costimulate T cells, together with the knowledge that the natural form of CD70 is a membrane-bound protein, firmly place CD27 in the TNFRSF category II group.

Despite having equivalent costimulatory activity to agonist anti-CD27 mAb *in vitro*, our initial evaluation of dtCD70-Fc *in vivo* suggested that the CD70 protein was less effective than anti-CD27 mAb in stimulating antigen-specific CD8 T cells (**Figure 2**). The difference in the activity between the two agents was particularly striking when the number of transferred OT-I T cells approximated the endogenous antigen-specific T cells (**Figure 2D**). Fc-fusion proteins are often cleared from the circulation more rapidly than antibodies due to several factors, including reduced affinity to FcRn and alterations in glycosylation (33). Although we did not detect differences in FcRn binding between dtCD70-Fc and anti-CD27 mAb (**Figure 3B**), glycan analysis demonstrated enrichment of oligomannose-type glycans in the dtCD70-Fc (**Figure 3C**, **Supplementary Figure 3**), which upon enzymatic removal improved its half-life (**Figure 3E**). As a result, the *in vivo* stimulatory activity of dtCD70-Fc was substantially enhanced and exceeded that of anti-CD27 mAb (**Figure 4A**, **Supplementary Figure 5**). We do not fully understand why dtCD70-Fc retains a high content of oligomannose-type glycans, but it is plausible that the presence of a large number (10) of N-linked glycans per polypeptide chain impacts on the efficiency of mannose trimming in the endoplasmic reticulum (45). Our data highlight the importance of glycan analysis when evaluating the *in vivo* behaviour of therapeutic glycoproteins and are consistent with previous studies on the role of oligomannose-type glycosylation in antibody and Fc-fusion protein clearance (32–34). Although our study did not reveal the identity of the receptor responsible for the rapid clearance of dtCD70-Fc, we speculate that this is largely mediated through uptake by the endocytic mannose receptor which is expressed on subpopulations of macrophages, dendritic cells and the hepatic sinusoidal endothelium. Previous work by Lee and colleagues (35) demonstrated that mannose receptor deficient mice exhibited a defect in the clearance of proteins bearing mannose or N-acetylglucosamine residues, highlighting the non-redundant role for this receptor in regulating glycoprotein half-life *in vivo*. Since two of the three N-glycosylation sites in the ECD of murine CD70 are conserved in human CD70, our findings will likely have relevance for the generation and use of human dtCD70-Fc.

Whilst the role of FcγRs in enhancing agonism by anti-TNFRSF antibodies is well established (36–38), to our knowledge this is the first demonstration that this phenomenon applies to soluble oligomeric CD70 (**Figure 4**, **Supplementary Figure 8**). However, unlike anti-CD27 mAb, dtCD70-Fc retained a significant proportion of its T cell stimulatory effects without the requirement of FcγR binding (**Figures 4**, **5**, **Supplementary Figure 8**). Previous studies have suggested that forced dimerization of soluble trimeric TNFSF ligands is required for activation of category II receptors (23, 46). Our data is consistent with this notion and additionally suggest that membrane association is required for maximal activity. In the current study, the Fc domain in dtCD70-Fc performed a dual function enforcing dimerization of CD70 trimers and tethering the protein to the plasma membrane of FcγR expressing cells. Further studies are required to assess if modulation of dtCD70-Fc binding to FcγR can be harnessed to tailor the magnitude of immune stimulation to the desired level and thus avoid a scenario whereby immune activation leads to an overt inflammatory response. In addition, it will be important in the future to understand how CD27 stimulation with or without engagement of FcγRs impact the differentiation and longevity of effector and memory T cell subsets.

In summary, we provide a method for the generation of a CD27 agonist with a tunable activity profile. The approach described here may encourage further exploration of TNFSF proteins in vaccine development and immunotherapy.

## Data availability statement

The original contributions presented in the study are included in the article/**Supplementary Material**. Further inquiries can be directed to the corresponding author.

## Ethics statement

The animal study was approved by UK Home Office licence numbers PA4C79999 and IE7C34E6C and following approval by the local ethics committee, reporting to the Home Office Animal Welfare Ethical Review Board (AWERB) at the University of Southampton. The study was conducted in accordance with the local legislation and institutional requirements.

## Author contributions

OD and CIM performed the experiments with the help of JK, HTCC, PJD, SLB and AR. JDA performed the site-specific glycan analysis. OD, JDA, SLB, CIM, AR, MC and AA-S analysed and interpreted the data. AA-S conceived the project. OD, JDA and AA-S wrote the manuscript with feedbacks from MC, SLB, AR and HTCC. All authors contributed to the article and approved the submitted version.
