# Supplementary material for: Fcγ receptor binding is required for maximal immunostimulation by CD70-Fc
Source: Front Immunol. 2023 Oct 27;14:1252274. doi: 10.3389/fimmu.2023.1252274 (PMC10641686; doi:10.3389/fimmu.2023.1252274)
Supplement: Supplementary file 1 [file DataSheet_1.pdf]

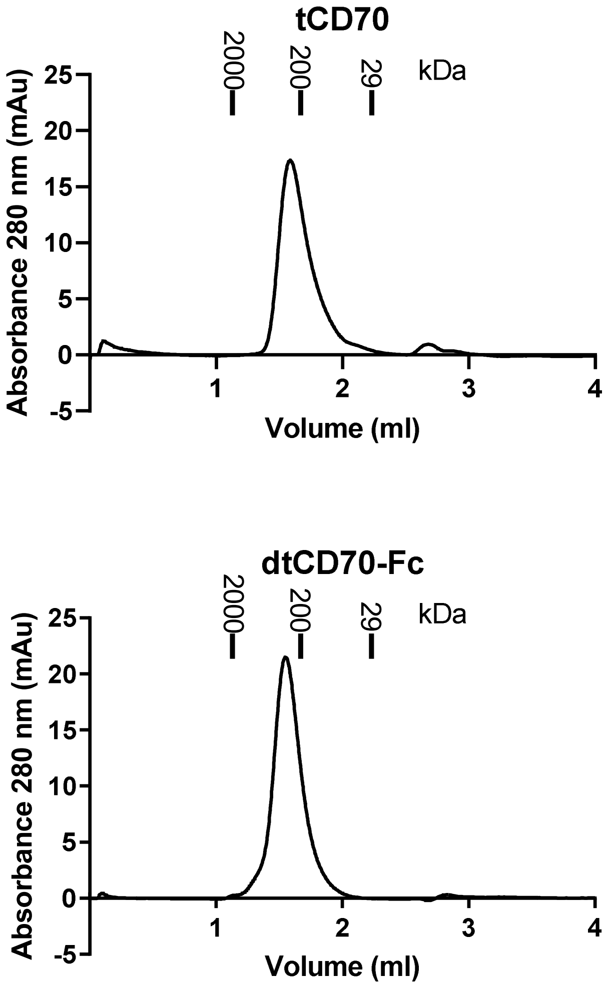

SUPPLEMENTARY FIGURE 1. Analytical SEC elution profiles of soluble CD70 fusion proteins (10 µg) obtained using a Superdex 200 5/150 GL size-exclusion column and PBS as an elution buffer.
