# Supplementary material for: Fcγ receptor binding is required for maximal immunostimulation by CD70-Fc
Source: Front Immunol. 2023 Oct 27;14:1252274. doi: 10.3389/fimmu.2023.1252274 (PMC10641686; doi:10.3389/fimmu.2023.1252274)
Supplement: Supplementary file 2 [file DataSheet_2.pdf]

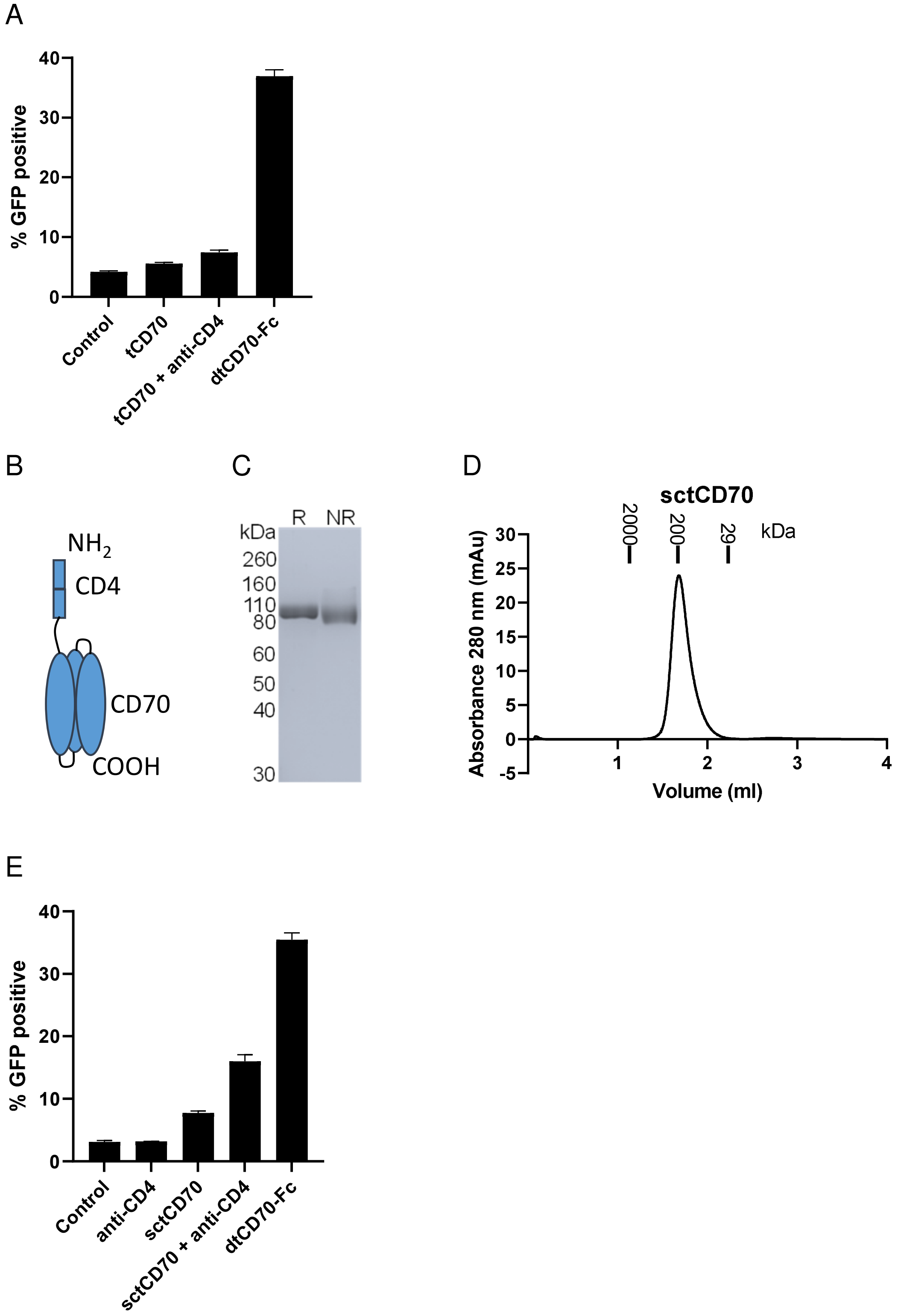

SUPPLEMENTARY FIGURE 2. Analysis of NFκB activation by CD70 fusion proteins. NFκB-GFP/mouse CD27^{+} Jurkat reporter cells were stimulated with of indicated proteins for 6 hrs at 37 °C before being analysed for GFP expression by flow cytometry. (A) The effect of tCD70 crosslinking using anti-CD4 on NFκB activation. (B) Schematic of the sctCD70 structure. (C and D) SDS-PAGE under reducing (R) and non-reducing (NR) conditions (C) and analytical SEC profile of sctCD70 protein (D). (E) The effect of sctCD70 crosslinking using anti-CD4 on NFκB activation. Data shown are the mean +/- SD. Controls were irrelevant mIgG1 in (A) and Jurkat reporter cells alone in (E).
