# Supplementary material for: Fcγ receptor binding is required for maximal immunostimulation by CD70-Fc
Source: Front Immunol. 2023 Oct 27;14:1252274. doi: 10.3389/fimmu.2023.1252274 (PMC10641686; doi:10.3389/fimmu.2023.1252274)
Supplement: Supplementary file 3 [file DataSheet_3.pdf]

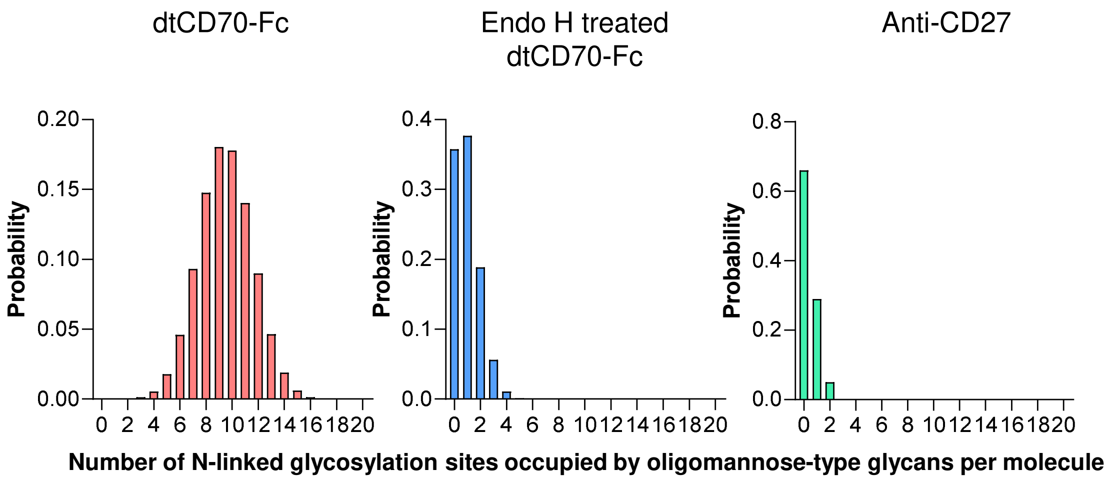

SUPPLEMENTARY FIGURE 3. Probability distribution of the number of potential N-linked glycosylation site occupied by oligomannose-type glycans per molecule. Using the average % oligomannose-type glycans calculated in Figure 3C, the distribution of the number of sites containing oligomannose-type glycans per molecule was calculated using the formula for binomial distribution.
