# Supplementary material for: Fcγ receptor binding is required for maximal immunostimulation by CD70-Fc
Source: Front Immunol. 2023 Oct 27;14:1252274. doi: 10.3389/fimmu.2023.1252274 (PMC10641686; doi:10.3389/fimmu.2023.1252274)
Supplement: Supplementary file 4 [file DataSheet_4.pdf]

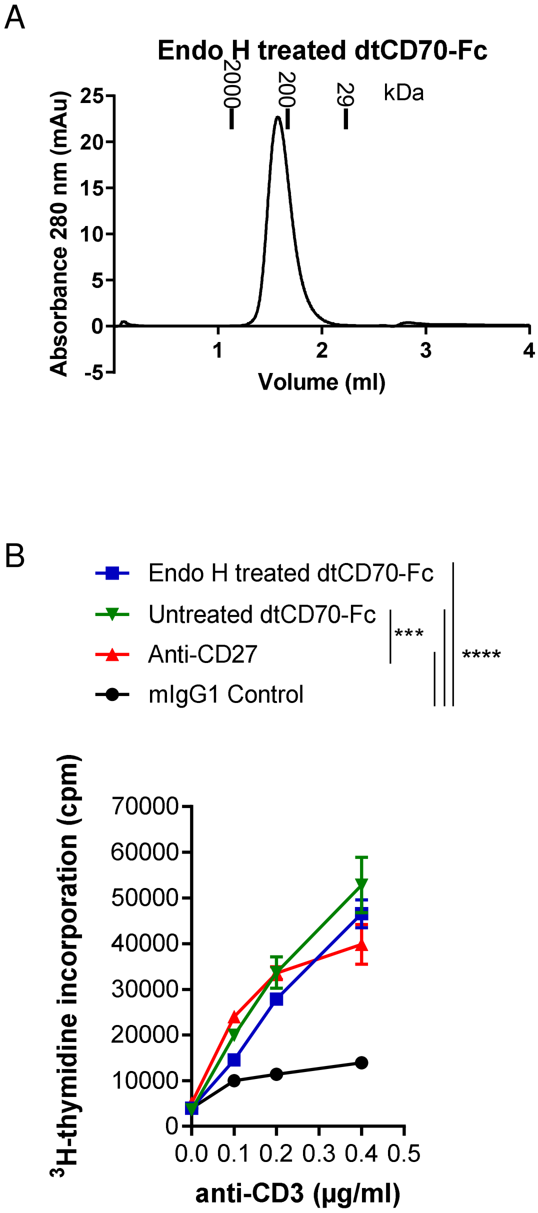

SUPPLEMENTARY FIGURE 4. Characterisation and in vitro activity of dtCD70-Fc following Endo H treatment. (A). Analytical SEC elution profile of Endo H treated dtCD70-Fc. (B) Endo H treated dtCD70-Fc exerts costimulatory effects similar to untreated dtCD70-Fc. Splenocytes were stimulated for 72 h with various concentrations of soluble anti-CD3 and the indicated proteins (10 μg/ml). Proliferation of T cells as assessed by measurement of [^{3}H]-thymidine incorporation. Data points represent the mean of triplicate measurements +/- SE and the data are representative of two independent experiments. Statistical comparisons at the highest anti-CD3 concentration are indicated. *** P < 0.001, **** P < 0.0001, two-way ANOVA with Tukey's multiple comparison test.
