# Supplementary material for: Fcγ receptor binding is required for maximal immunostimulation by CD70-Fc
Source: Front Immunol. 2023 Oct 27;14:1252274. doi: 10.3389/fimmu.2023.1252274 (PMC10641686; doi:10.3389/fimmu.2023.1252274)
Supplement: Supplementary file 5 [file DataSheet_5.pdf]

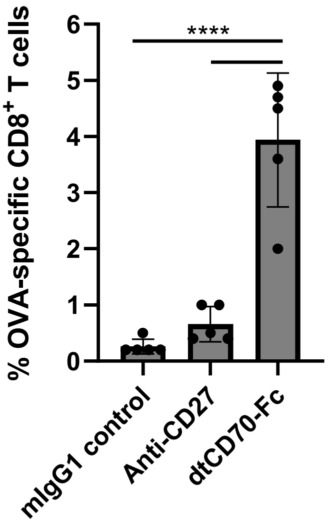

SUPPLEMENTARY FIGURE 5. Analysis of endogenous $OVA_{257\text{-}264}$ specific $CD8^+$ T cell response. Mice were injected i.v. with OVA protein (5 mg) in combination with mIgG1 control, anti-CD27 or Endo H treated dtCD70-Fc (250 µg) on day 0. Mice received 2 further injections of mIgG1/anti-CD27/dtCD70-Fc on days 1 and 2. The percentages of $OVA_{257\text{-}264}$ specific $CD8^+$ T cells out of total $CD8^+$ T cells were determined in blood on day 7 by tetramer staining and flow cytometry. Data shown represent mean +/- SD (n=5 mice/group). **** $P < 0.0001$, one-way ANOVA with Tukey's multiple comparisons test.
