# Supplementary material for: Fcγ receptor binding is required for maximal immunostimulation by CD70-Fc
Source: Front Immunol. 2023 Oct 27;14:1252274. doi: 10.3389/fimmu.2023.1252274 (PMC10641686; doi:10.3389/fimmu.2023.1252274)
Supplement: Supplementary file 6 [file DataSheet_6.pdf]

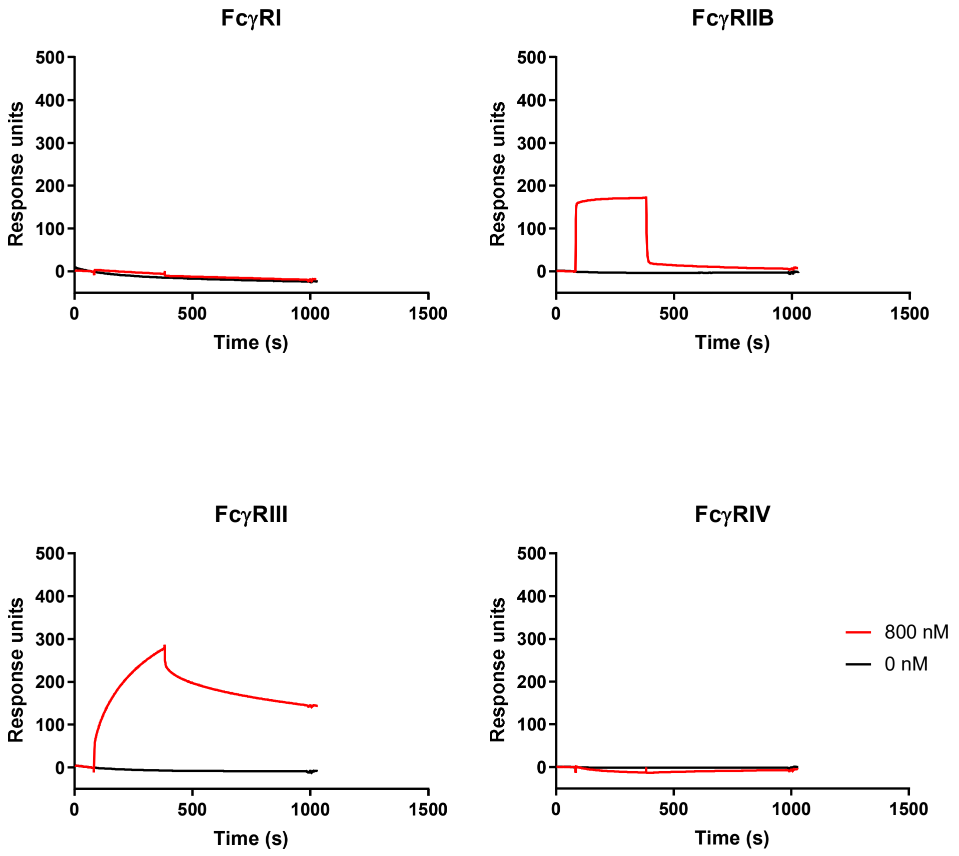

SUPPLEMENTARY FIGURE 6. SPR analysis of the binding of Endo H treated dtCD70-Fc to FcγRs. Soluble recombinant FcγRs (800 nM) were injected over immobilised Endo H treated dtCD70-Fc (~3000 RU) for 5 mins at a flow rate of 30 µl/min. Sensograms show measurable binding to FcγRIIB and FcγRIII, consistent with the binding specificity of mIgG1 Fc.
