# Supplementary material for: Fcγ receptor binding is required for maximal immunostimulation by CD70-Fc
Source: Front Immunol. 2023 Oct 27;14:1252274. doi: 10.3389/fimmu.2023.1252274 (PMC10641686; doi:10.3389/fimmu.2023.1252274)
Supplement: Supplementary file 7 [file DataSheet_7.pdf]

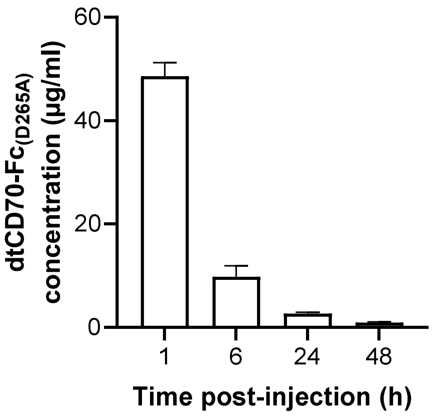

SUPPLEMENTARY FIGURE 7. The concentrations of dtCD70-$Fc_{(D265A)}$ in serum samples (n = 3) were measured by ELISA at the indicated intervals following i.v. injection (250 µg).
