# Supplementary material for: Fcγ receptor binding is required for maximal immunostimulation by CD70-Fc
Source: Front Immunol. 2023 Oct 27;14:1252274. doi: 10.3389/fimmu.2023.1252274 (PMC10641686; doi:10.3389/fimmu.2023.1252274)
Supplement: Supplementary file 8 [file DataSheet_8.pdf]

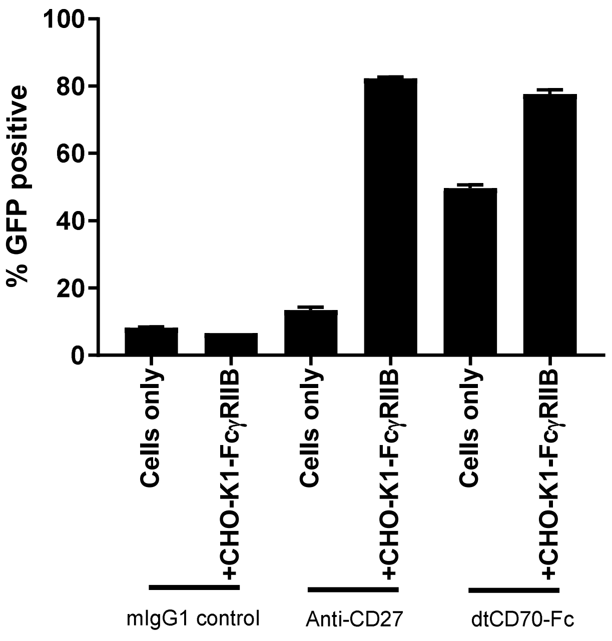

SUPPLEMENTARY FIGURE 8. Crosslinking of anti-CD27 and dtCD70-Fc by FcγRIIB promotes their activity. NFκB-GFP/mouse $CD27^+$ Jurkat reporter cells were stimulated with indicated proteins without (cells only) or in the presence of CHO-K1 cells that express mouse FcγRIIB for 6 hrs at 37 °C before being analysed for GFP expression by flow cytometry. Data shown are the mean +/- SD.
